# MEG Insight into the Spectral Dynamics Underlying Steady Isometric Muscle Contraction

**DOI:** 10.1523/JNEUROSCI.0447-17.2017

**Published:** 2017-10-25

**Authors:** Mathieu Bourguignon, Harri Piitulainen, Eero Smeds, Guangyu Zhou, Veikko Jousmäki, Riitta Hari

**Affiliations:** ^1^Department of Neuroscience and Biomedical Engineering and Aalto NeuroImaging, Aalto University School of Science, 00076 AALTO, Espoo, Finland,; ^2^Basque Center on Cognition, Brain and Language, 20009 San Sebastian, Spain,; ^3^Laboratoire de Cartographie fonctionnelle du Cerveau, Neurosciences Institute, Université libre de Bruxelles, 1070 Brussels, Belgium,; ^4^Department of Neurology, Feinberg School of Medicine, Northwestern University, Chicago, Illinois 60611,; ^5^NatMEG, Karolinska Institutet, 171 77 Stockholm, Sweden,; ^6^Cognitive Neuroimaging Centre, Lee Kong Chian School of Medicine, Nanyang Technological University, Singapore 636921, and; ^7^Department of Art, Aalto University School of Arts, Design, and Architecture, 00076 AALTO, Helsinki, Finland

**Keywords:** cortex-muscle coherence, corticokinematic coherence, isometric contraction, magnetoencephalography, motor control, primary sensorimotor cortex

## Abstract

To gain fundamental knowledge on how the brain controls motor actions, we studied in detail the interplay between MEG signals from the primary sensorimotor (SM1) cortex and the contraction force of 17 healthy adult humans (7 females, 10 males). SM1 activity was coherent at ∼20 Hz with surface electromyogram (as already extensively reported) but also with contraction force. In both cases, the effective coupling was dominant in the efferent direction. Across subjects, the level of ∼20 Hz coherence between cortex and periphery positively correlated with the “burstiness” of ∼20 Hz SM1 (Pearson *r* ≈ 0.65) and peripheral fluctuations (*r* ≈ 0.9). Thus, ∼20 Hz coherence between cortex and periphery is tightly linked to the presence of ∼20 Hz bursts in SM1 and peripheral activity. However, the very high correlation with peripheral fluctuations suggests that the periphery is the limiting factor. At frequencies <3 Hz, both SM1 signals and ∼20 Hz SM1 envelope were coherent with both force and its absolute change rate. The effective coupling dominated in the efferent direction between (1) force and the ∼20 Hz SM1 envelope and (2) the absolute change rate of the force and SM1 signals. Together, our data favor the view that ∼20 Hz coherence between cortex and periphery during isometric contraction builds on the presence of ∼20 Hz SM1 oscillations and needs not rely on feedback from the periphery. They also suggest that effective cortical proprioceptive processing operates at <3 Hz frequencies, even during steady isometric contractions.

**SIGNIFICANCE STATEMENT** Accurate motor actions are made possible by continuous communication between the cortex and spinal motoneurons, but the neurophysiological basis of this communication is poorly understood. Using MEG recordings in humans maintaining steady isometric muscle contractions, we found evidence that the cortex sends population-level motor commands that tend to structure according to the ∼20 Hz sensorimotor rhythm, and that it dynamically adapts these commands based on the <3 Hz fluctuations of proprioceptive feedback. To our knowledge, this is the first report to give a comprehensive account of how the human brain dynamically handles the flow of proprioceptive information and converts it into appropriate motor command to keep the contraction force steady.

## Introduction

Steady muscle contraction is maintained by a continuous drive from the cortex to spinal motoneurons and by fine motor adjustments according to proprioceptive feedback (Scott, 2012). Still, we do not know how the brain integrates this proprioceptive feedback to affect motor control.

During steady contraction, the sensorimotor cortical rhythms and the muscle activity, as measured with surface EMG, are coupled at ∼20 Hz, a phenomenon known as cortex–muscle coherence (Conway et al., 1995; Salenius et al., 1996, 1997). Some authors suggested that cortex–muscle coherence reflects cortical drive to the muscles (Salenius et al., 1997; Gross et al., 2000), whereas others argued for the existence of a reafferent contribution (Riddle and Baker, 2005; Baker, 2007; Witham et al., 2011) used for sensorimotor control (Baker, 2007). According to this latter view, ∼20 Hz sensorimotor oscillations could represent a cortical state that promotes the maintenance of steady motor output (Gilbertson et al., 2005; Androulidakis et al., 2006, 2007; Baker, 2007; Witham et al., 2011). Alternatively, according to a hypothetic mechanism, the sensorimotor system could send pulses at ∼20 Hz and monitor the resulting afferent signal to probe the state of the periphery for continuous sensorimotor recalibration (Mackay, 1997; Baker, 2007). Still, these accounts of the functional role of ∼20 Hz reafferent signals are highly speculative.

During transient limb movements, cortex–muscle coherence disappears (Kilner et al., 2000) and primary sensorimotor (SM1) activity is coupled with movement kinematics (Kelso et al., 1998; O'Suilleabhain et al., 1999; Jerbi et al., 2007; Bourguignon et al., 2011, 2012; Piitulainen et al., 2013a, b). This coupling is known as the corticokinematic coherence (CKC). When the movement is regular, CKC mainly peaks at movement frequency and its first harmonic (Bourguignon et al., 2011, 2012; Piitulainen et al., 2013a, b). Irregular movements lead to CKC at frequencies <8 Hz (Jerbi et al., 2007). The slow brain activity underpinning CKC is typically strong and reliable enough for brain machine interfaces to use them to detect some features of the movement (Hammon et al., 2008; Waldert et al., 2008; Bradberry et al., 2010; Jerbi et al., 2011). However, CKC appears to reflect the processing of proprioceptive feedback generated by the movements. Indeed, voluntary and passive movements elicit similar CKC level (Piitulainen et al., 2013a), and directionality analyses revealed that the afferent component dominates over the efferent one (Bourguignon et al., 2015).

CKC also unfolds during slow tracking movements. Indeed, the kinematics of slow tracking movements is characterized by fluctuations at frequencies of 1–4 Hz (Craik, 1947), regardless of movement speed (Miall et al., 1986; Roitman et al., 2004; Pasalar et al., 2005), and these fluctuations synchronize with SM1 activity (Dipietro et al., 2011; Hall et al., 2014). In that context, rhythmic fluctuations in movement position are termed submovements (Craik, 1947).

Here, we tested the hypothesis that CKC also manifests itself during isometric steady muscle contractions. To that aim, we analyzed the coupling between MEG signals (Harri and Puce 2017) and natural force fluctuations in an isometric contraction task. First, we expected to uncover significant coupling at ∼20 Hz since a previous study revealed the existence of ∼20 Hz coherence between MEG and acceleration signals recorded from the index finger in an isometric wrist extension task (Airaksinen et al., 2015). We expected that coupling to dominate in the efferent direction, as is the case for ∼20 Hz coupling with EMG. Second, we expected to uncover significant coupling between MEG signals and unavoidable slight fluctuations in contraction force at frequencies <3 Hz (where rhythmic fluctuations in the applied force are the strongest). We expected that coupling to dominate in the afferent direction, as is the case during voluntary movements.

## Materials and Methods

### 

#### Subjects

Seventeen healthy human volunteers (7 females, 10 males; mean ± SD age, 34 ± 7 years; age range, 20–47 years) with no history of neuropsychiatric diseases or movement disorders participated in our study. All subjects were right-handed (mean ± SD score, 90 ± 12; range, 65–100 on the scale from −100 to 100; Edinburgh handedness inventory) (Oldfield, 1971).

The study had a prior approval by the ethics committee of the Aalto University. The subjects gave informed consent before participation, and they were compensated monetarily for travel expenses and lost working hours.

#### Experimental protocol

**Figure 1.**
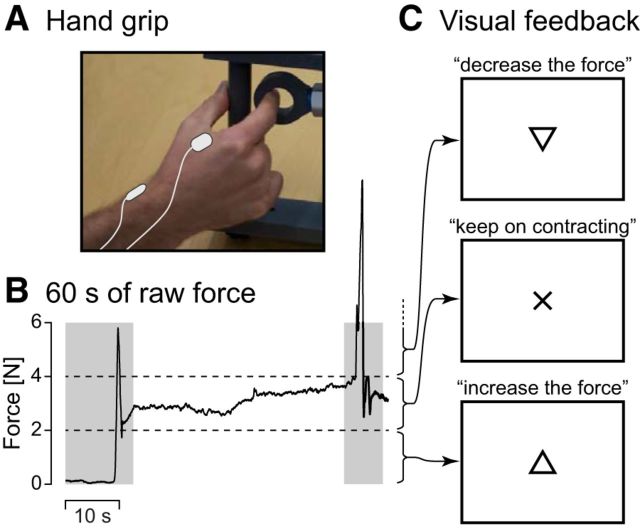
Experimental setup. ***A***, Illustration of the isometric contraction task. A steady contraction is maintained on a custom-made handgrip with the right thumb and the index finger. Surface EMG is measured from the first dorsal interosseous (top right electrode) and the flexor carpi ulnaris (not visible here) of the right hand, with reference electrode over the distal radial bone (bottom left electrode). ***B***, Sixty seconds of raw force signal from a representative subject. The subject was prompted to start contracting 10 s after the beginning of the recording. Two horizontal dashed lines indicate the force limits (2–4 N). Gray shadowed areas represent periods wherein contraction force was out of the prescribed bounds for at least 1 time-bin 2 s around. Corresponding data were not analyzed. ***C***, Visual feedback presented to the subjects to help them regulate their contraction force. Cross on the screen informed them that the force was within the prescribed limits. Arrow pointing up (respectively down) prompted them to increase (respectively decrease) the force.

Figure 1 illustrates the experimental setup. During MEG recordings, the subjects were sitting with their left hand on the thigh and their right hand on a table in front of them. Subjects' vision was optically corrected with nonmagnetic goggles (Medigoggles, Cambrigde Research Systems) when needed. Subjects were asked to maintain a steady isometric pinch grip of 2–4 N against a custom-made handgrip (connected to a rigid load cell; rigidity 15.4 N/mm; model 1004, Vishay Precision Group) with the right thumb and the index finger (Fig. 1*A*), and to fixate at a black cross displayed on the center of a screen placed 1 m away in front of them. When the force stepped out of the prescribed limits, a triangle (pointing up or down) appeared on top of the black cross, indicating in which direction to adjust the force, and it disappeared as soon as the force was correctly returned within the limits (Fig. 1*B*,*C*). After a ∼1 min practice session, two 5 min blocks were recorded, with a minimum of 2 min rest between the blocks. Each block started with ∼10 s without contraction, after which subjects were prompted to begin the contraction task. A 5 min task-free block was recorded as well.

#### Measurements

##### MEG.

The MEG measurements were performed in a three-layer magnetically shielded room (Imedco AG at the MEG Core of Aalto NeuroImaging; http://ani.aalto.fi), Aalto University, with a 306-channel whole-scalp neuromagnetometer (Elekta Neuromag, Elekta Oy). The recording passband was 0.1–330 Hz, and the signals were sampled at 1 kHz. Subjects' head position inside the MEG helmet was continuously monitored by feeding current into four head-tracking coils located on the scalp; the locations of the coils and at least 200 head-surface points (scalp and nose) with respect to anatomical fiducials were determined with an electromagnetic tracker (Fastrak, Polhemus).

##### EMG and force.

Surface EMG was measured from the first dorsal interosseous and the flexor carpi ulnaris of the right hand, which are both recruited during a pinch grip task. EMG electrodes were placed on the muscle bulk, and signals were measured with respect to an electrode placed over the distal radial bone. Recording passband was 10–330 Hz for EMG signals and DC–330 Hz for the force signal. EMG and force signals were then sampled at 1 kHz and recorded time-locked to MEG signals.

##### MRI.

3D-T1 MRIs were acquired with Signa 3.0 T whole-body MRI scanner (Signa VH/i, General Electric) or with 3T MAGNETOM Skyra whole-body MRI scanner (Siemens Healthcare) at the AMI Centre, Aalto NeuroImaging, Aalto University School of Science.

#### Data preprocessing

Continuous MEG data were preprocessed off-line with MaxFilter 2.2.10 (Elekta Oy), including head movement compensation. The tSSS preprocessing was applied with a correlation limit of 0.9 and segment length equal to the recording length (Taulu and Kajola, 2005; Taulu and Simola, 2006). Independent component analysis was then applied to MEG signals filtered through 1–25 Hz, and 1–3 components corresponding to eye-blink and heartbeat artifacts were visually identified based on their topography and time-series. The corresponding components were subsequently subtracted from raw MEG signals.

#### Functional (nondirectional) coupling

We used coherence analysis to estimate the functional coupling between MEG and all peripheral (2 EMGs, force) signals, and to identify the optimal MEG sensor and muscle for further analyses. Time periods coinciding with visual feedback (i.e., when the force level was not properly kept between 2 and 4 N) were marked as bad to exclude periods during which contraction was intentionally corrected. Time periods for which MEG signals exceeded 5 pT (magnetometers) or 1 pT/cm (gradiometers) were also marked as bad to avoid contamination of the data by any artifact not removed by the preprocessing. Continuous data from the recording blocks were split into 1000 ms epochs with 800 ms epoch overlap (Bortel and Sovka, 2014), leading to a frequency resolution of 1 Hz. Epochs <2 s away from timepoints marked as bad were discarded from further analyses (mean ± SD artifact-free epochs, 2875 ± 130; range, 2573–3031). Coherence spectra were computed between all MEG sensors and nonrectified EMG signals and force signal following the formulation of Halliday et al. (1995), and by using the multitaper approach (5 orthogonal Slepian tapers, yielding a spectral smoothing of ±2.5 Hz) to estimate power- and cross-spectra (Thomson, 1982). Data from gradiometer pairs were combined in the direction of maximum coherence as done by Bourguignon et al. (2015). Then, for each subject and reference signal, the gradiometer pair with the highest coherence value in the 10–30 Hz band was selected among a predefined subset of 9 gradiometer pairs covering the left rolandic region.

A threshold for statistical significance of the coherence (*p* < 0.05 corrected for multiple comparisons) was obtained as the 95th percentile of the distribution of the maximum coherence (across 10–30 Hz, and across the same 9 gradiometers) evaluated between MEG and Fourier transform surrogate reference signals (1000 repetitions) (Faes et al., 2004). The Fourier transform surrogate of a signal is obtained by computing its Fourier transform, replacing the phase of the Fourier coefficients by random numbers in the range (−π; π), and then computing the inverse Fourier transform (Theiler et al., 1992; Faes et al., 2004). Further analyses were performed for each reference signal with the selected gradiometer signal in the orientation yielding the maximum coherence (MEG_SM1_). Finally, as EMG was recorded from 2 synergistic muscles, we analyzed only the muscle showing the highest coherence in the 10–30 Hz range (first dorsal interosseous for 10 subjects and flexor carpi ulnaris for the remaining 7 subjects).

Detailed investigations revealed that the ∼20 Hz coherence with the force was dampened due to spectral leakage of lower frequencies that were substantially higher in amplitude (see Fig. 1*B*). For this reason, the coherence was reevaluated based on a procedure less sensitive to spectral leakage as described below. MEG and peripheral signals were filtered through 5-Hz-wide frequency bands centered on 5–40 Hz by steps of 1 Hz. The bandpass filter used in that effect was designed in the frequency domain with zero-phase and 1-Hz-wide squared-sine transitions from 0 to 1 and 1 to 0 (e.g., the filter centered on 20 Hz rose from 0 at 17 Hz to 1 at 18 Hz and ebbed from 1 at 22 Hz to 0 at 23 Hz). Of note, MEG signals were smoothly set to 0 (squared-cosine transition of 1 s) at timings 1 s around artifacts (timepoints of MEG signals exceeding 5 pT for magnetometers or 1 pT/cm for gradiometers); and to avoid edge effects, further analyses were based on timepoints at least 2 s away from artifacts and appearance of visual feedback. The analytical signals were then created by means of the Hilbert transform. From these analytical signals (*s_k_*(*f*, *t*); *k* = 1, 2 indexing the MEG and the peripheral signal respectively), amplitude spectra were estimated as follows:

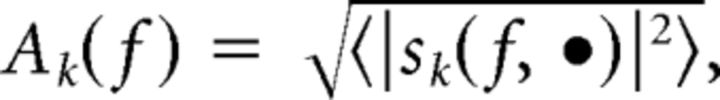
 where |·| denotes the absolute value and 〈·〉 the average across time. Coherence was then estimated as follows:

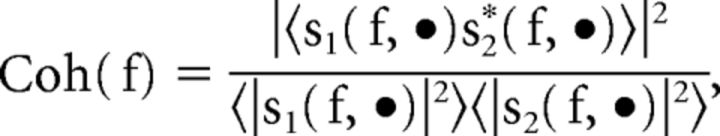
 where a superscript * denotes the complex conjugate. This procedure consistently enhanced the coherence with the force signal while it left the coherence with EMG signals virtually unchanged.

**Figure 2.**
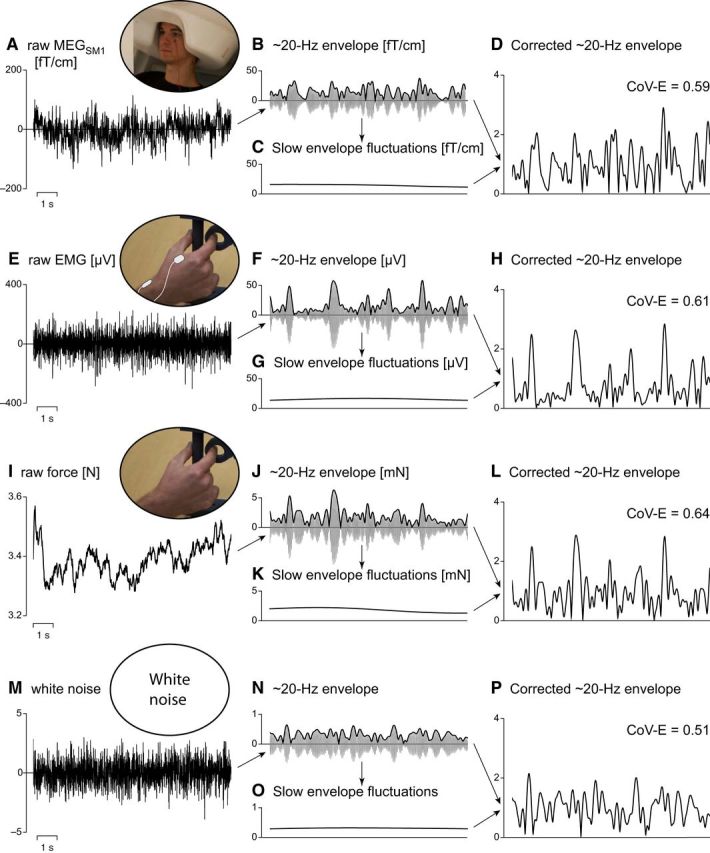
Illustration of the procedure used to obtain envelopes of band-limited signals corrected for slow drifts. ***A***, Raw MEG signal recorded over the primary sensorimotor cortex (MEG_SM1_). ***B***, Band-limited MEG_SM1_ envelope. That is, the Hilbert envelope (black trace) of MEG_SM1_ signal filtered through a narrow band (gray trace). In the present illustration, the band was 5 Hz wide and centered on 20 Hz (hence the ∼20 Hz MEG_SM1_ envelope). The same procedure was repeated for center frequencies from 5 to 40 Hz by steps of 1 Hz (data not shown on the figure). ***C***, <0.1 Hz fluctuations of the envelope displayed in ***B***. ***D***, Envelope corrected for slow drifts. That is, the envelope (displayed in ***B***) divided timepoint by timepoint by its slow fluctuations (displayed in ***C***). The coefficient of variation (CoV) of that envelope (CoV-E; computed over 10 min) is indicated in the top right corner. ***E–H***, Same as ***A–D*** for the EMG signal. ***I–L***, Same as ***A–D*** for the force signal. ***M–P***, Same as ***A–D*** for a white noise. All corrected envelopes fluctuate around 1, but qualitatively less so for the white noise, leading to a lower CoV.

Finally, we also estimated envelope correlation. To avoid contamination by very slow envelope variations, both envelope signals (|*s_k_*(*f*, *t*)|, *k* = 1, 2) were divided by their low-pass filtered version at 0.1 Hz (squared-sine transition from 0.05 to 0.15 Hz). This procedure is illustrated in Figure 2. Such normalization ensured that the correlation was blind to changes slower than 0.1 Hz that could be linked to, for example, changes in contraction strategy (which induces substantial changes in EMG amplitude). Hence, the envelope coupling was estimated as follows:

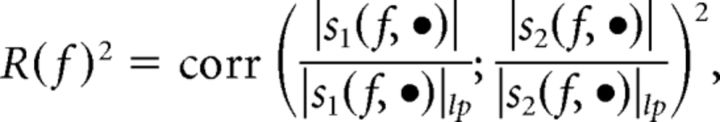
 where the subscript “lp” stands for “low pass filtered at 0.1 Hz.”

The same analysis was repeated with a finer spectral resolution for the force signal only. In this analysis, amplitude and coherence spectra were computed based on MEG and force signals filtered through 0.6-Hz-wide frequency bands centered on 0.4–40 Hz by steps of 0.2 Hz.

#### Effective (directional) coupling

We used renormalized partial directed coherence (rPDC) (Schelter et al., 2006, 2009) to estimate the causal influence of force and MEG_SM1_ signals on one another. The estimation of the rPDC requires fitting a multivariate autoregressive model to the data, and the parameters of the model determine the frequency resolution. Here, a multivariate autoregressive model of order 100 was fitted to the data low-pass filtered at 50 Hz and resampled at 100 Hz with the ARfit package (Schneider and Neumaier, 2001). Across subjects and conditions, the optimal model order range was 17–34 (mean ± SD, 21 ± 4) according to Schwarz's Bayesian criterion and 46–376 (166 ± 97) according to Akaike's final prediction error, both implemented in the ARfit package (Schneider and Neumaier, 2001). Adopting a model order of 100 therefore represented a good compromise between the two criteria. The chosen parameters (resampling and model order) enabled us to explore frequencies up to 50 Hz with a 1 Hz frequency resolution. Finally, to achieve a frequency smoothing similar to that of coherence spectra, rPDC was smoothed with a square kernel wide of 5 frequency bins, following the approach proposed by Sommerlade et al. (2009). The ensuing spectral smoothing was ±2.5 Hz. This procedure yielded for each subject one rPDC spectrum in the efferent direction (MEG_SM1_ → force) and one in the afferent direction (force → MEG_SM1_).

#### Link between cortex–muscle coherence and burstiness of brain and peripheral signals

We performed additional analyses to clarify the causal influence of ∼20 Hz fluctuations in SM1 and peripheral signals on one another. We know that activities of both the SM1 cortex and periphery (EMG, force) are characterized by bursts of ∼20 Hz cycles often followed by ∼1-s-long silent periods (Jasper and Penfield, 1949; Murthy and Fetz, 1992, 1996; Baker et al., 1997; Gilbertson et al., 2005). We also know that the ∼20 Hz cortex–muscle coherence is tightly linked to the presence of these ∼20 Hz bursts in both SM1 and peripheral signals (McAuley et al., 1997; Kilner et al., 2000, 2003; Gilbertson et al., 2005; Kristeva et al., 2007; Ushiyama et al., 2011; Matsuya et al., 2013). Accordingly, we here quantify the “burstiness” of MEG_SM1_ and peripheral signals, and strive to ascribe the interindividual variability in this burstiness to that in ∼20 Hz coherence between MEG_SM1_ and peripheral signals. Of note, the magnitude of the cortex–muscle coherence is widely known for its great interindividual variability, from values below the detection limit afforded by even 10-min-long recordings (i.e., ∼<0.003) up to values of 0.3 (Pohja et al., 2005; Bayraktaroglu et al., 2013).

Figure 2 illustrates the processing procedure we used (detailed below) to estimate the “burstiness” of MEG_SM1_ and peripheral signals. The coefficient of variation (CoV; ratio between the SD and the mean) of the envelope (CoV-E) of a bursting signal is higher than that of white noise filtered similarly (in our setting, the CoV-E of a filtered white noise is ∼0.51). Accordingly, we filtered MEG_SM1_ and peripheral signals through 5-Hz-wide frequency bands centered on 5–40 Hz by steps of 1 Hz, and computed their CoV-E. To avoid contamination by very slow amplitude variations, amplitude signals were divided by their low-pass filtered version at 0.1 Hz before estimating the CoV.

A threshold for statistical significance of maximal CoV-E value across 10–30 Hz (*p* < 0.05 corrected for multiple comparisons, one threshold per subject and per signal: MEG_SM1_, force, and EMG) was obtained as the 95th percentile of the distribution of the maximum CoV-E, across 10–30 Hz, of white noise signals (1000 repetitions) (Faes et al., 2004).

We searched for an association between (1) the magnitude of ∼20 Hz cortex–muscle coherence (maximum across 10–30 Hz) and (2) the CoV-E of ∼20 Hz MEG_SM1_ and peripheral signals (maximum across 10–30 Hz) with Spearman and Pearson correlation (across the 17 subjects). Pairs of Pearson correlation coefficients were compared with the Steiger test (Steiger, 1980).

We also computed the sensor topography for the CoV-E of ∼20 Hz MEG signals. Practically, for each gradiometer pair, we retained the maximum CoV-E of the MEG signals (1) in 20 different directions (from 0 to π) within the 2D space spanned by the two gradiometers, and (2) filtered through 5-Hz-wide frequency bands centered on 18–22 Hz by steps of 1 Hz.

#### Coupling at lower frequencies

**Figure 3.**
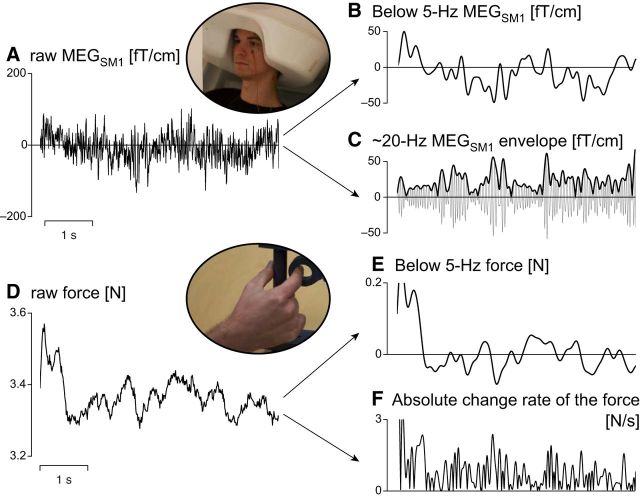
Representative samples of brain and force signals between which <3 Hz coupling was sought. ***A***, Raw MEG_SM1_. ***B***, MEG_SM1_ signal low-pass filtered at 5 Hz. ***C***, The ∼20 Hz MEG_SM1_ envelope. That is, the Hilbert envelope (black trace) of the MEG_SM1_ signal filtered through 15–25 Hz (gray trace). ***D***, Raw force signal during a period of stable contraction (same as in ***A***). ***E***, Force signal low-pass filtered at 5 Hz. ***F***, The absolute change rate of the force. That is, the force signal (1) bandpass filtered through 0.5–10 Hz, (2) differentiated, and (3) rectified.

Figure 3 presents representative samples of the slow fluctuations of MEG_SM1_ and force signals considered here.

We estimated the coherence at lower frequencies (<5 Hz) between MEG signals (Fig. 3*B*) and force fluctuations (Fig. 3*E*). The analysis was identical to that described in Coherence analysis, except that epochs were 5000 ms long, affording a finer spectral resolution of 0.2 Hz and 3 tapers were used (yielding a spectral smoothing of ±0.3 Hz). In addition, MEG and force signals were high-pass filtered at 0.2 Hz to avoid spectral leakage from near to DC components. Another difference was that the threshold for statistical significance (*p* < 0.05 corrected for multiple comparisons) was obtained for the mean coherence across 0.5–3 Hz.

The same analysis was repeated to evaluate the coupling between MEG signals and fluctuations in the absolute change rate of the force (Fig. 3*F*). The absolute change rate of the force was obtained as the absolute value of the time derivative of the force signal band-passed through 0.5–10 Hz. It is relevant to seek coupling with this signal given that some proprioceptive receptors (e.g., Golgi tendon organs) are sensitive to the force itself, whereas others (e.g., the primary endings of muscle spindles) are sensitive to the change rate of muscle stretch.

The same analysis was repeated also to evaluate the coupling between the envelope of MEG signals filtered through 15–25 Hz (Fig. 3*C*) and the slow fluctuations of both the force signal and its absolute change rate. In what follows, the envelope of MEG signals filtered through 15–25 Hz will be referred to as the ∼20 Hz MEG envelope. This envelope signal fluctuates at frequencies <10 Hz.

We used rPDC to quantify the causal influence of the following: (1) the envelope of ∼20 Hz MEG_SM1_ signals, (2) the MEG_SM1_ signals as such, and (3) the force on one another. A multivariate autoregressive model of order 50 was fitted to the data low-pass filtered at 5 Hz and resampled at 10 Hz, enabling us to explore frequencies up to 5 Hz with a 0.2 Hz frequency resolution. Across subjects and conditions, the optimal model order range was 2–6 (mean ± SD, 4 ± 1.5) according to Schwarz's Bayesian criterion and 59–147 (96 ± 25) according to Akaike's final prediction error. To achieve a frequency smoothing similar to that of coherence, rPDC was smoothed with a square kernel wide of 3 frequency bins, leading to a spectral smoothing of ±0.3 Hz. This procedure yielded, for each subject, 6 rPDC spectra: one for each possible combination of directions (2 possibilities) and signal pairs (3 possibilities).

The same procedure was repeated to quantify the causal influence of the following: (1) the envelope of ∼20 Hz MEG_SM1_ signals, (2) the MEG_SM1_ signals as such, and (3) the absolute change rate of the force on one another.

Finally, we used temporal response functions (TRFs) to model how force signals affect the temporal dynamics of ∼20 Hz MEG_SM1_ envelope and MEG_SM1_ signals. A similar approach has been used previously to model brain responses to continuous speech sounds (Lalor and Foxe, 2010; Zion Golumbic et al., 2013; Lawler et al., 2015). TRFs are the direct analog of evoked responses in the context of continuous stimulation.

Practically, we used the mTRF toolbox (Crosse et al., 2016) to estimate the TRF of MEG_SM1_ and of ∼20 Hz MEG_SM1_ envelope associated with force signals, all signals being filtered through 0.5–5 Hz and downsampled to 20 Hz. For each subject, the TRFs were modeled from −1.5 to 2.5 s, for a fixed set of ridge values (λ = 2^0^, 2^1^, 2^2^… 2^20^). We adopted the following 10-fold cross-validation procedure to determine the optimal ridge value: For each subject, TRFs were estimated based on 90% of the data. TFRs were then multiplied by a window function with squared-sine transition on the edges (rising from 0 at −1.5 s to 1 at 1 s and ebbing from 1 at 2 s to 0 at 2.5 s) to dampen regression artifacts. We next used these windowed TRFs to predict the 10% of data left out, and estimated the Pearson correlation coefficient between predicted and measured signals. These correlation values in the 10 runs were tested against 0 with a Wilcoxon signed rank test. Finally, the square of the mean correlation value across the 10 runs provided an estimate of the proportion of variance explained by the signals imputable to force fluctuations. In the final analysis, we used the ridge value maximizing the mean explained variance across our 17 subjects (sum of the logarithm across the 2 signals considered: MEG_SM1_, and ∼20 Hz MEG_SM1_ envelope; λ = 2^15^).

The same procedure was repeated to estimate the TRFs to the absolute change rate of the force of MEG_SM1_, and ∼20 Hz MEG_SM1_ envelope, all signals being filtered through 0.5–5 Hz.

#### Impact of heartbeats on low-frequency coupling

Heartbeats transiently increase blood pressure in all blood vessels of the body, leading to changes in the pressure measured at the fingertips (Parati et al., 1989). Such transient pressure changes in the fingertips are thus expected to slightly impact the contraction force at frequencies matching cardiac rhythm and its harmonics, and they also produce some heartbeat-locked changes in the proprioceptive afference because of modified muscle spindle firing (Birznieks et al., 2012). Also, electric activity of the heart generates magnetocardiographic artifacts in MEG signals (Jousmäki and Hari, 1996). We therefore estimated the impact of heartbeats on both force and brain signals.

We estimated the TRF of individual subjects' force and brain signals associated with heartbeats. Because ECG was not recorded in the present study, we recovered the heartbeats from unprocessed magnetometer signals. In practice, we performed an ICA decomposition of all the 102 magnetometer signals and retained the cardiac component based on visual inspection of the time course and topography of each independent component. Based on these ECG artifacts, we identified the timing of the QRS complexes (mainly R peaks) to generate a virtual signal that takes value 1 at R peaks and 0 elsewhere. TRFs of force, MEG_SM1_, and ∼20 Hz MEG_SM1_ envelope associated with this R-timing signal were estimated as described in the previous paragraph (λ = 2^12^), with the only exception that TRFs were modeled from −1 to 2 interheartbeat intervals.

Furthermore, we used partial coherence to evaluate the degree of coupling between force and brain signals controlled for heartbeats. Partial coherence should not be confounded with rPDC. Partial coherence is the direct extension of partial correlation to the frequency domain. It provides an estimate of the coherence between two signals while removing the linear contribution of a third signal, i.e., it controls for that contribution (Halliday et al., 1995). Here, partial coherence was obtained with the same parameters as those described in Coupling at lower frequencies, between force and brain signals (both MEG and ∼20 Hz MEG envelope) while removing the linear contribution of the R-timing signal.

Finally, to gain more insight into the physiological role of heartbeats on the coupling between force and brain signals, we compared the coherence at frequencies closest to individual heart rate and its harmonics (f_heart-rate_) with that at frequencies farthest from heart rate and its harmonics (f_off-heart-rate_). To that aim, and for each subject, we estimated the minimum distance from each frequency bins in 0.5–3 Hz to heart rate and its harmonics. The first half of the frequency bins of minimum distance were assigned f_heart-rate_ and the other half to f_off-heart-rate._ For each subject, we then evaluated the mean coherence at f_heart-rate_ and f_off-heart-rate_ separately and compared both measures between subjects with a Wilcoxon test.

#### Source reconstruction

We estimated the cortical distribution of the coherence phenomena highlighted by the sensor-level analyses. To that aim, individual MRIs were first segmented using Freesurfer software (Martinos Center for Biomedical Imaging, Charlestown, MA; RRID:SCR_001847) (Reuter et al., 2012). MEG and segmented MRI coordinate systems were coregistered using the three anatomical fiducial points for initial estimation and the head-surface points to manually refine the surface coregistration. Then, the MEG forward model based on a one-shell boundary element model of the intracranial space was computed for three orthogonal current dipoles placed on a homogeneous 5 mm grid source space that covered the whole brain (MNE suite; Martinos Center for Biomedical Imaging, Charlestown, MA; RRID:SCR_005972) (Gramfort et al., 2014). To simultaneously combine data from the planar gradiometer and the magnetometer sensors for source estimation, sensor signals and the corresponding forward-model coefficients were normalized by their root-mean-square noise estimated from the task-free data filtered through 1–195 Hz. For each source, the forward model was then reduced to its two principal components of highest singular value, which closely correspond to sources tangential to the skull. Source time courses were then estimated from the sensor data with a minimum-variance beamformer built with the covariance matrix of the task-free data filtered through 1–195 Hz (Van Veen et al., 1997). Maps of the parameters estimated in the sensor space were estimated similarly in the source space (source pairs taking the role of the gradiometer pairs). Then, a nonlinear transformation from individual MRIs to the standard MNI brain was computed using the spatial-normalization algorithm implemented in Statistical Parametric Mapping (SPM8; Wellcome Department of Cognitive Neurology, London; RRID:SCR_007037) (Ashburner et al., 1997; Ashburner and Friston, 1999) and applied to individual maps. Finally, group-level maps were obtained by averaging normalized maps across subjects.

## Results

### Coupling with EMG and force at ∼20 Hz

**Figure 4.**
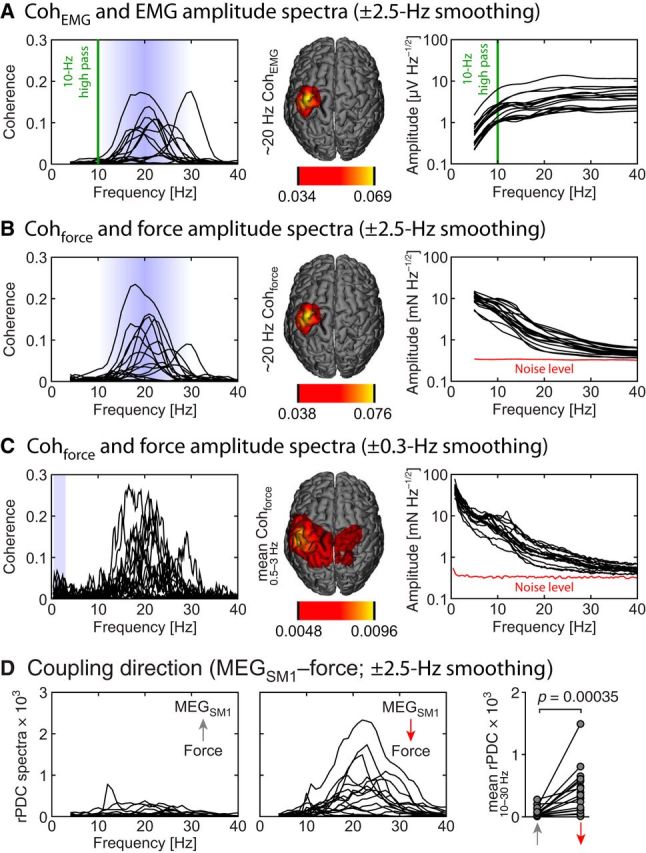
Coupling between cortical signals and the periphery. ***A***, Left to right, Individual coherence between MEG_SM1_ and EMG signals (Coh_EMG_) with ±2.5 Hz smoothing (one trace per subject; *n* = 17), corresponding group-average source map at ∼20 Hz, and EMG amplitude spectra. The EMG signals were high-pass filtered at 10 Hz (green vertical line). ***B***, Same as ***A*** with EMG replaced by force. Red line indicates the noise level of the force signals. ***C***, Same as ***B*** but with ±0.3 Hz spectral smoothing and group-average source maps computed at 0.5–3 Hz. ***D***, Directional coupling between MEG_SM1_ and force quantified with rPDC.

MEG oscillations measured from the contralateral (left) SM1 cortex (MEG_SM1_) were coherent at ∼20 Hz with the surface EMG (Coh_EMG_; Fig. 4*A*; Table 1) as is widely known in the literature (Conway et al., 1995; Salenius et al., 1996, 1997) but also with the fluctuations of the contraction force (Coh_force_; Fig. 4*B*; Table 1). Coh_EMG_ and Coh_force_ were statistically significant in all subjects (*p* values <0.05; surrogate-data-based statistics), and their maximum values were highly correlated (*r* = 0.90, *p* < 0.0001; Spearman correlation). The peak frequencies and maximum values of Coh_EMG_ and Coh_force_ did not differ statistically significantly from each other (*p* = 0.11 and *p* = 0.068, respectively; Wilcoxon test). These results are in line with a previous finding that, during static extension of the wrist, SM1 activity is coherent at 20 Hz with finger vibrations recorded with an accelerometer (Airaksinen et al., 2015).

**Table 1.** Maximum Coh_force_ and Coh_EMG_ values across 10–30 Hz and corresponding peak frequencies (mean, SD, and range across subjects)

10–30 Hz coherence	Force		EMG	
	Mean ± SD	Range	Mean ± SD	Range
Coherence strength	0.083 ± 0.067	0.0033–0.235	0.072 ± 0.058	0.0030–0.175
Peak frequency [Hz]	19.6 ± 4.7	11–29	20.0 ± 5.1	10–29

The existence of ∼20 Hz Coh_force_ implies that steady contractions inherently involve ∼20 Hz force fluctuations that are coherent with ∼20 Hz SM1 oscillations. A close inspection of the force amplitude spectra (Fig. 4*B*) revealed clear peaks at ∼10 Hz, the typical frequency of physiological tremor (McAuley et al., 1997; Gilbertson et al., 2005), but not at ∼20 Hz (for amplitude values, see Table 2). The existence of genuine ∼20 Hz tremor has nevertheless been demonstrated previously by showing its synchronization with ∼20 Hz MEG_SM1_ signals (Airaksinen et al., 2015), which was evident also in the current study, and by relating finger ∼20 Hz tremor to impairment in motor performance (McAuley et al., 1997; Gilbertson et al., 2005).

**Table 2.** Amplitude of force fluctuations at 1 and 20 Hz (mean, SD, and range across subjects)

Force amplitude	At 1 Hz		At 20 Hz	
	Mean ± SD	Range	Mean ± SD	Range
Force [mN Hz^−1/2^]	40 ± 10	25–61	1.2 ± 0.5	0.5–2.2

A natural question to ask is whether the sensorimotor system is sensitive enough to detect reafferent signals associated with the nonsalient ∼20 Hz force fluctuations (Fig. 4*B*,*C*). Our response is a definitive yes because the Golgi tendon organs can detect stretches as low as 30–90 μN during active muscle contraction (Binder et al., 1977) and thus are sensitive enough to detect the force fluctuations of 2900 ± 1200 μN we observed in the current study (values integrated over a 5 Hz band from 17.5 to 22.5 Hz). Nevertheless, the directional coupling strength was 5.6 times stronger in the efferent than in the afferent direction, as quantified by rPDC (mean across 10–30 Hz; Fig. 4*D*; *p* = 0.00035, Wilcoxon test), implying that ∼20 Hz Coh_force_ mainly reflects effects of the efferent cortical drive, similarly to what has been demonstrated for cortex–muscle coherence (Salenius et al., 1997; Gross et al., 2000; Witham et al., 2011; Lim et al., 2014).

### Link between cortex-muscle coherence and burstiness of brain and peripheral signals

**Figure 5.**
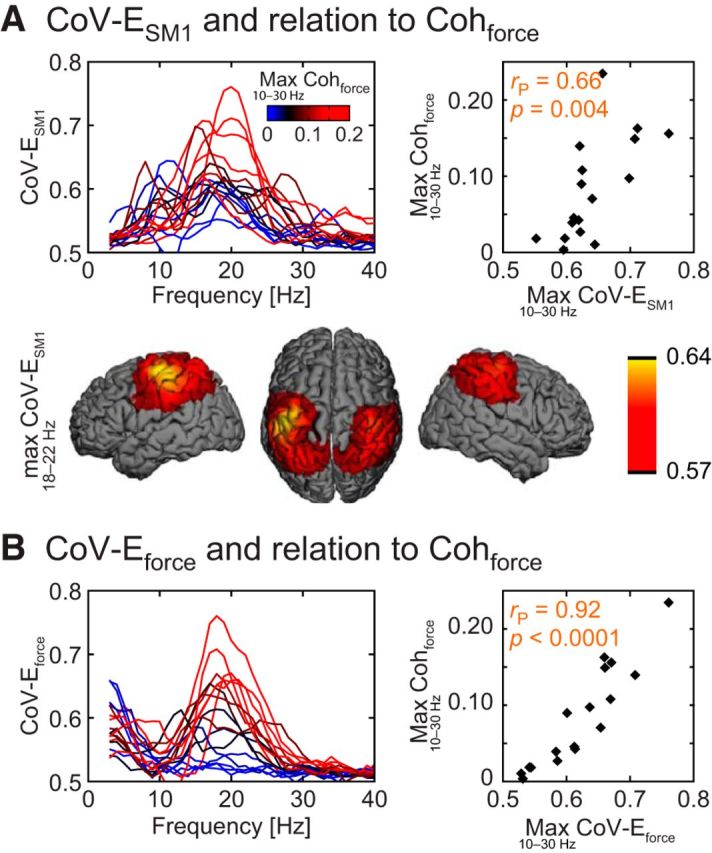
“Burstiness” of MEG_SM1_ and force signals, and relation with ∼20 Hz Coh_force_. ***A***, Left, CoV-E_SM1_ as a function of the carrier frequency. Individual traces (*n* = 17) are color-coded for their corresponding maximal Coh_force_ in the 10–30 Hz range. CoV-E_SM1_ peaks for carrier frequencies at ∼20 Hz with a magnitude apparently linked to ∼20 Hz Coh_force_. Right, Relation between the maximum values of CoV-E_SM1_ and Coh_force_ across the 10–30 Hz range. Top left, Pearson correlation coefficient (*r*_P_) and corresponding significance level. Bottom, Surface rendering of the group-averaged CoV-E of ∼20 Hz source-projected MEG signals. ***B***, Same as ***A*** for CoV-E_force_. CoV-E_force_ peaks for carrier frequencies at ∼20 Hz with a magnitude directly linked to ∼20 Hz Coh_force_.

**Figure 6.**
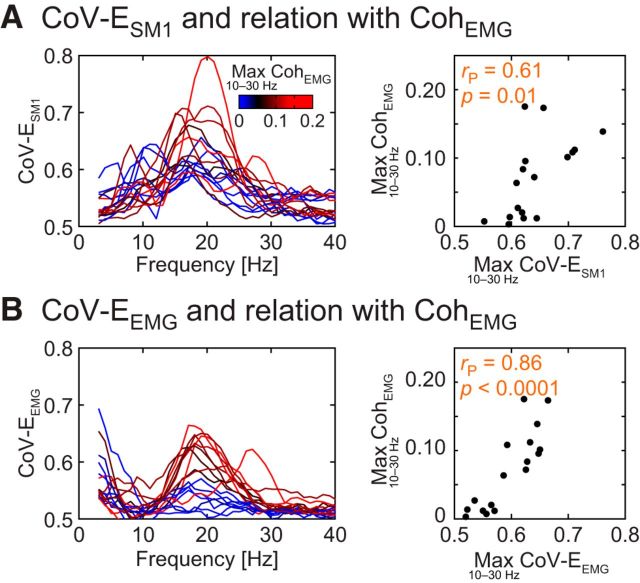
“Burstiness” of MEG_SM1_ and EMG signals, and relation with ∼20 Hz Coh_EMG_. ***A***, Left, CoV-E_SM1_ as a function of the carrier frequency. Individual traces (*n* = 17) are color-coded for their corresponding maximal Coh_EMG_ in the 10–30 Hz range. CoV-E_SM1_ peaks for carrier frequencies at ∼20 Hz with a magnitude apparently linked to ∼20 Hz Coh_EMG_. Some CoV-E_SM1_ traces in this figure differ from those in Figure 5*A* because they were obtained from different MEG_SM1_ signals (i.e., MEG_SM1_ signal was selected to either maximize Coh_EMG_ or Coh_force_ at ∼20 Hz). ***A***, Right, Relation between the maximum values of CoV-E_SM1_ and Coh_EMG_ across the 10–30 Hz range. Top left, Pearson correlation coefficient (*r*_P_) and corresponding significance level. ***B***, Same as ***A*** for CoV-E_EMG_. CoV-E_EMG_ peaks for carrier frequencies at ∼20 Hz with a magnitude directly linked to ∼20 Hz Coh_EMG_.

Here we relate the interindividual variability in the burstiness of brain and peripheral signals (quantified with CoV-E) to that in Coh_force_ and Coh_EMG_ to draw conclusions about the causal influence of ∼20 Hz brain and peripheral signals on one another. The CoV-E of MEG_SM1_ (CoV-E_SM1_), EMG (CoV-E_EMG_), and force (CoV-E_force_) peaked for carrier signal at ∼20 Hz, as was observed in all subjects for CoV-E_SM1_ (Figs. 5*A*, 6*A*) and in the majority of the subjects for CoV-E_force_ (Fig. 5*B*) and CoV-E_EMG_ (Fig. 6*B*). Indeed, CoV-E_SM1_, CoV-E_EMG_, and CoV-E_force_ peak values in 10–30 Hz exceeded significantly the ∼0.51 level expected for white noise for all subjects (*p* < 0.05), except for 2 subjects for CoV-E_EMG_. Importantly, the ∼20 Hz peak of individual subjects' CoV-E_SM1_, CoV-E_force_, and CoV-E_EMG_ tightly matched in all subjects, except in these 2 subjects lacking significant CoV-E_EMG_, therefore supporting physiological rather than artifact origins of these envelope modulations.

**Figure 7.**
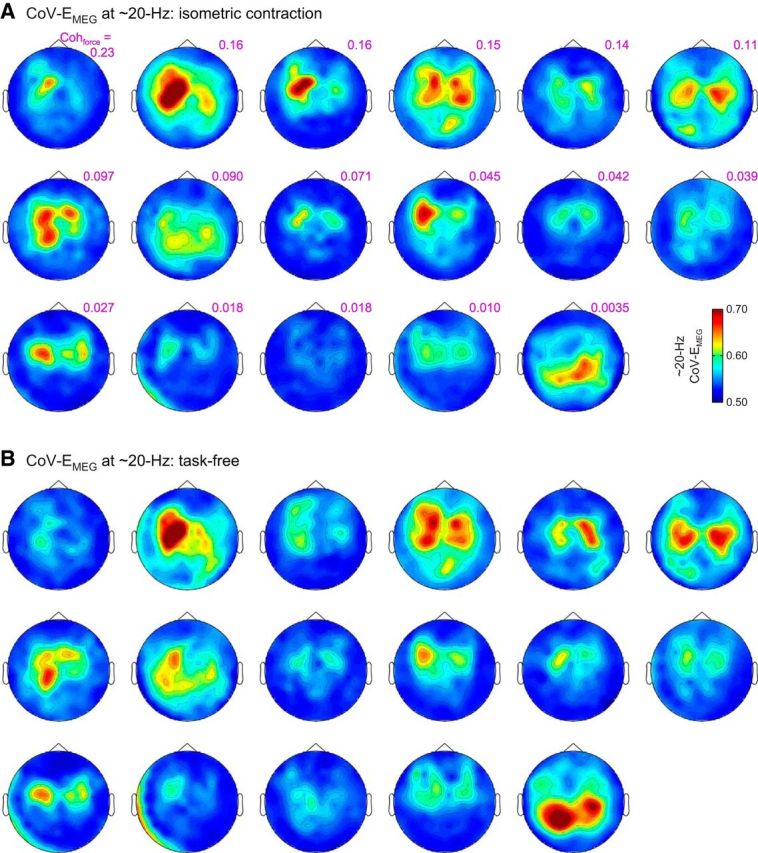
Sensor topography of the CoV of the envelope of ∼20 Hz MEG signals (CoV-E_MEG_) during the isometric contraction task (***A***) and during the task-free session (***B***). Subjects are ordered according to their maximum coherence between MEG and force signals (Coh_force_) across 10–30 Hz.

Moreover, the spatial pattern of the 20 Hz CoV-E_SM1_ agreed with the origin of the signal variations in the rolandic region (Figs. 5*A*, 7*A*). Of note, the corresponding topography obtained with task-free MEG data was similar (Fig. 7*B*), and so were the ∼20 Hz CoV-E_SM1_ values (*p* = 0.83; isometric contraction vs task-free; Wilcoxon test).

**Figure 8.**
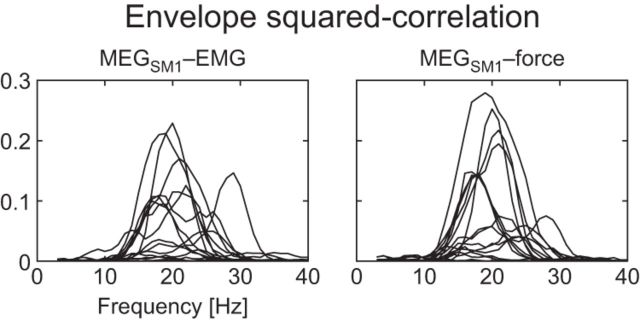
Envelope squared-correlation of MEG_SM1_ with peripheral signals (EMG and force; one trace per subject; *n* = 17). The correlation was computed between the envelopes of the signals filtered through a 5-Hz-wide bands centered on frequencies from 5 to 40 Hz by steps of 1 Hz.

As last confirmation of the physiological origin of ∼20 Hz envelope fluctuations, the ∼20 Hz bursts in MEG_SM1_ and peripheral signals occur in synchrony. This can be appreciated from the raw envelope traces displayed in Figure 2*D*, *H*, *L*. More quantitatively, MEG_SM1_-peripheral envelope correlation peaked for carrier frequencies of ∼20 Hz in the majority of our subjects (Fig. 8). This was expected since significant envelope correlation was previously reported for ∼20 Hz MEG_SM1_ and EMG signals (Bayraktaroglu et al., 2013).

Across subjects, the maximum CoV-E_SM1_ across the 10–30 Hz range correlated with the maximum coherence across the same range (Spearman correlation; Coh_force_, *r* = 0.75, *p* = 0.0008, see Fig. 5*A*; Coh_EMG_, *r* = 0.73, *p* = 0.0012, see Fig. 6*A*), but the association was not strong (Pearson correlation; Coh_force_, *r* = 0.66, *p* = 0.004; Coh_EMG_, *r* = 0.61, *p* = 0.01); some subjects with almost identical CoV-E_SM1_ had coherence values differing by a factor of ∼10. The maximum CoV-E_force_ and CoV-E_EMG_ across the 10–30 Hz range correlated with the corresponding maximum coherences across the same range (Spearman correlation; Coh_force_, *r* = 0.93, *p* < 0.0001, see Fig. 5*B*; Coh_EMG_, *r* = 0.81, *p* = 0.0001, see Fig. 6*B*), and the association was strong (Pearson correlation: force, *r* = 0.92, *p* < 0.0001; EMG, *r* = 0.86, *p* < 0.0001). Indeed, the association with the coherence magnitude was significantly stronger with CoV-E_force_ than with CoV-E_SM1_ (*z* = 2.47, *p* = 0.013; Steiger test), and marginally stronger with CoV-E_EMG_ than with CoV-E_SM1_ (*z* = 1.87, *p* = 0.061).

**Figure 9.**
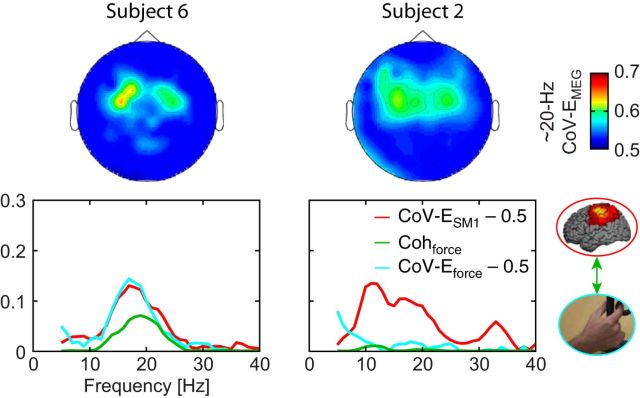
Limiting factor in ∼20 Hz cortex–muscle coherence: illustration with 2 representative subjects. Top, Sensor topography of the CoV of MEG envelope (CoV-E_MEG_) for carrier frequency of ∼20 Hz. Bottom, CoV-E_SM1_ (red traces), CoV-E_force_ (cyan traces), and Coh_force_ (green traces) as function of the frequency. Subject 6 displayed ∼20 Hz bursting activity in MEG_SM1_ and force signals that led to average coherence level. In comparison, Subject 2 displayed similar ∼20 Hz bursting activity in MEG_SM1_ (similar CoV-E_MEG_ sensor topography and CoV-E_SM1_ traces) but highly reduced CoV-E_force_ and Coh_force_. None of the subjects displayed the reversed pattern (similar CoV-E_force_ and reduced CoV-E_SM1_ and Coh_force_).

These results confirm that ∼20 Hz cortex–muscle coherence is tightly linked to the presence of ∼20 Hz bursts in MEG_SM1_ and peripheral signals; but ultimately, the periphery appears to be the limiting factor. Indeed, some subjects with elevated magnitude of MEG_SM1_ bursts had low coherence, whereas, in contrast, subjects with low (respectively high) CoV-E_force_ had systematically low (respectively high) Coh_force_. This relationship is illustrated in Figure 9 where two subjects with similar ∼20 Hz CoV-E_SM1_ have strikingly different ∼20 Hz Coh_force_ while their CoV-E_force_ and Coh_force_ vary hand in hand. We take these findings as evidence that, in ∼20 Hz cortex–muscle coherence, the bursting SM1 activity drives the periphery and the ∼20 Hz bursts are transmitted with a subject-dependent efficiency.

### <3 Hz coupling with force

**Figure 10.**
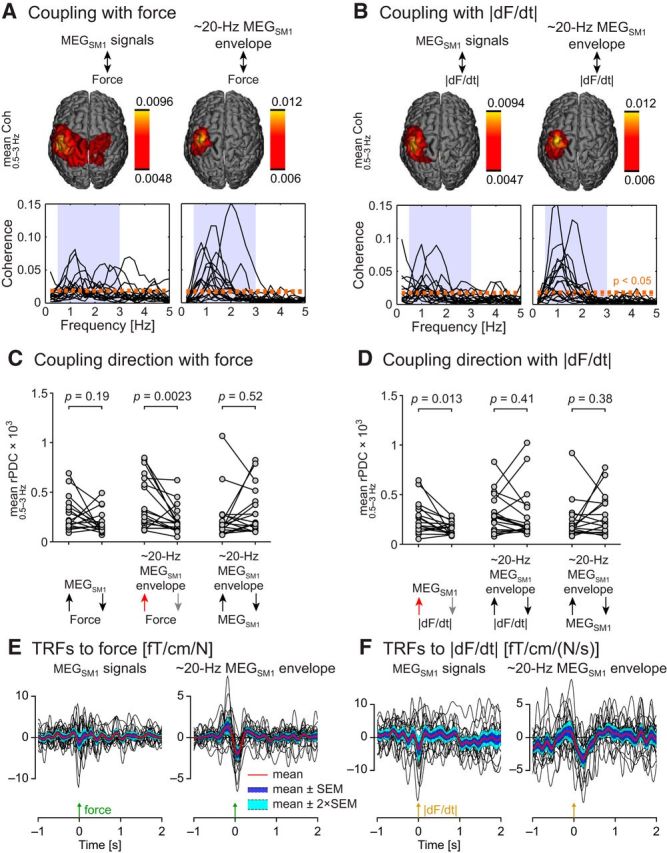
Coupling of MEG_SM1_ with <3 Hz force fluctuations. ***A***, Coherence between force and MEG_SM1_ signals (left), and between force and ∼20 Hz MEG _SM1_ envelope (right). Sub-blocks display the group-averaged coherence source map at 0.5–3 Hz (top) and the individual coherence spectra with the MEG_SM1_ signals. For indicative purpose, an orange dotted line indicates the significance level of the mean coherence across 0.5–3 Hz. ***B***, Same as in ***A*** with the force replaced by its absolute change rate (|dF/dt|). ***C***, ***D***, Directional coupling quantified with renormalized partial directed coherence (rPDC). ***E***, ***F***, TRFs associated to force (***E***) and |dF/dt| (***F***) of <5 Hz MEG_SM1_ (left) and ∼20 Hz MEG_SM1_ envelope (right). The plots display individual TRFs (black traces) as well as the mean TRF across subjects (red trace) ±1 SEM (blue area) and ±2 SEM (cyan area). Note that we reversed the polarity of TRFs of MEG_SM1_ signals (when the scalar product with the first principal component of sensor orientation was negative) to ensure that all TRFs were estimated in a compatible orientation. This was necessary to warrant the validity of the across-subjects averaging procedure.

Although peaking at ∼20 Hz, Coh_force_ was also salient <3 Hz (Fig. 4*C*). This frequency range corresponds to the strongest bulk of force fluctuations. Indeed, the power of force fluctuations is ∼35 times stronger at 1 Hz than at 20 Hz (Fig. 4*C*; Table 2). A dedicated coherence analysis revealed that both (1) ∼20 Hz MEG_SM1_ envelope and (2) MEG_SM1_ signals were significantly coherent with both force (Fig. 10*A*) and its absolute change rate (Fig. 10*B*) in 13–16 subjects of 17 (*p* < 0.05; mean coherence across 0.5–3 Hz; Table 3).

**Table 3.** Mean Coh_force_ across 0.5–3 Hz and corresponding peak frequencies (mean, SD, and range across subjects)*^a^*

0.5–3 Hz coherence	Force		Absolute change rate of the force	
	Mean ± SD	Range	Mean ± SD	Range
Coherence strength				
∼20 Hz MEG_SM1_ envelope	0.023 ± 0.017	0.005–0.073	0.021 ± 0.015	0.006–0.055
Slow MEG_SM1_ activity	0.019 ± 0.008	0.007–0.032	0.017 ± 0.009	0.007–0.037
Peak frequency [Hz]				
∼20 Hz MEG_SM1_ envelope	1.27 ± 0.41	0.8–1.8	1.13 ± 0.27	0.6–1.6
Slow MEG_SM1_ activity	1.64 ± 0.67	0.8–2.6	1.26 ± 0.52	0.6–2.4

*^a^*Values reported here were taken from the gradiometer pair of maximum mean Coh_force_ for each subject separately.

**Figure 11.**
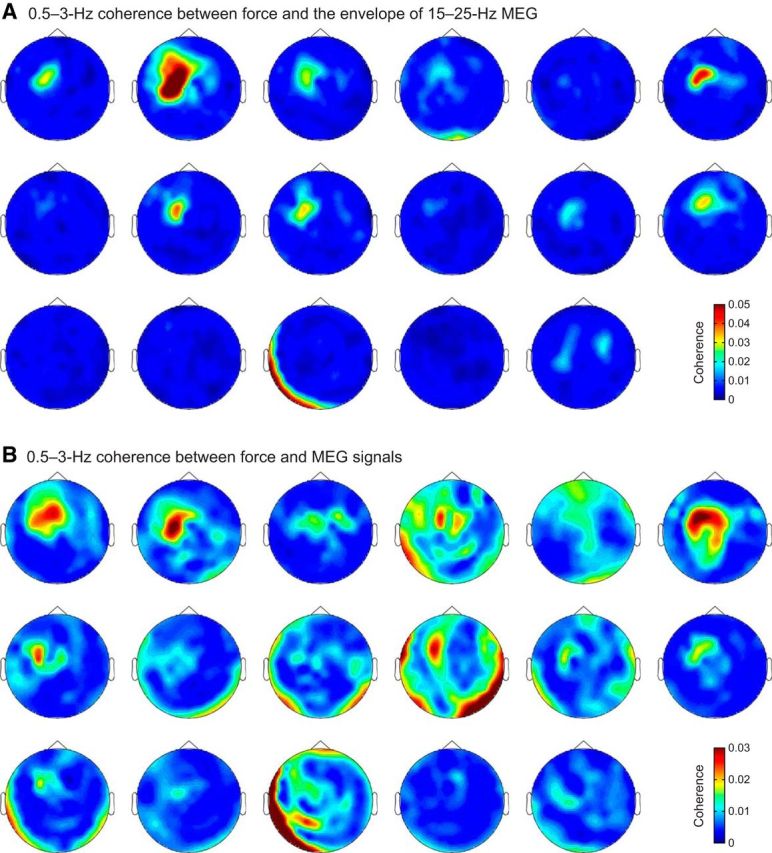
Sensor topography for the 0.5–3 Hz coherence of the force with (***A***) the envelope of 15–25 Hz MEG and (***B***) MEG signals. Subjects are ordered as in Figure 7.

**Figure 12.**
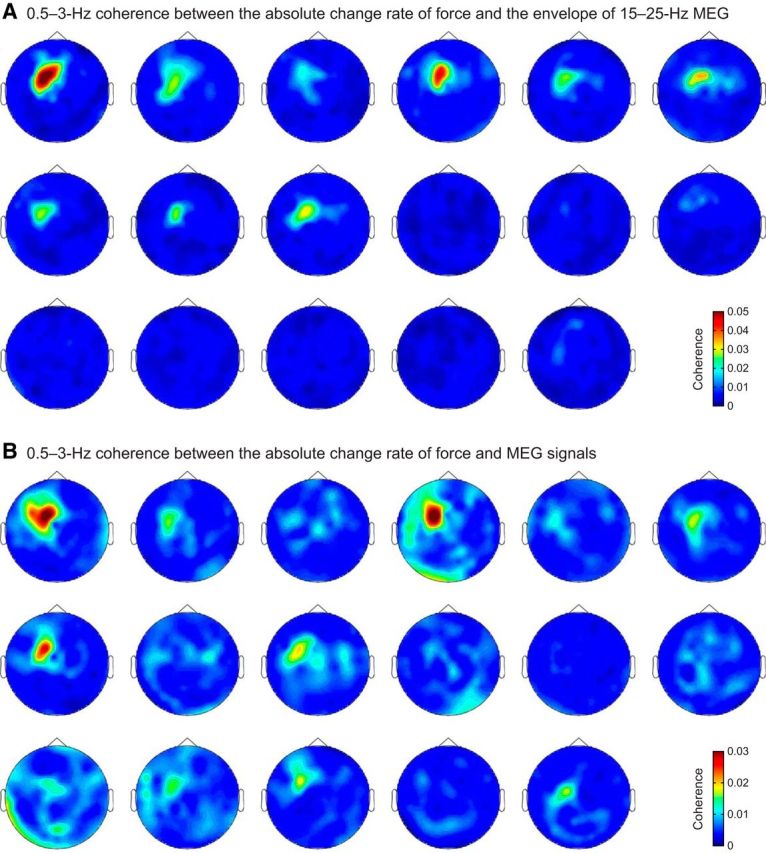
Sensor topography for the 0.5–3 Hz coherence of the absolute change rate of the force with (***A***) the envelope of 15–25 Hz MEG and (***B***) MEG signals. Subjects are ordered as in Figure 7.

The MEG signals related to these four investigated couplings originated from the SM1 cortex, as was confirmed by individual coherence maps (Figs. 11, 12) and by group-level source-space maps (Fig. 10*A*,*B*). At the SM1 sensor with highest mean coherence, the coherence peaked at frequencies between 0.6 and 2.4 Hz (Fig. 10*A*,*B*; Table 3).

Directionality analyses revealed a significantly stronger afferent than efferent coupling (quantified with the mean rPDC across 0.5–3 Hz) between (1) force fluctuations and the ∼20 Hz MEG_SM1_ envelope (*p* = 0.0023; Wilcoxon test; Fig. 10*C*), and (2) the absolute change rate of the force and the MEG_SM1_ signals (*p* = 0.013; Wilcoxon test; Fig. 10*D*). No statistically significant differences in the mean rPDC were found for the other pairs of signals (*p* values >0.05).

We further characterized the dynamics of brain signals with respect to changes in force using the TRFs (Fig. 10*E*,*F*). Table 4 shows the percentage of variance explained by TRFs. TRFs of the ∼20 Hz MEG_SM1_ envelope associated with force consistently decreased within 100 ms following the force signal (group-level minimum at 60 ms) and showed weaker, although still prominent, increase peaking ∼200 ms before the force signal. Individual TRFs of MEG_SM1_ signals associated with force consistently showed their highest values around time 0, but the phase of the oscillations was inconsistent across subjects. Similar observations can be made on TRFs associated with the absolute change rate of the force, but this time, the maximum decrease of ∼20 Hz MEG_SM1_ envelope occurred later (peak at 220 ms).

**Table 4.** Percentage of variance explained by temporal response functions (mean, SD, and range across subjects, and number of subjects in whom it was statistically significant)

Variance explained	Mean ± SD [%]	Range [%]	*n*
By force			
MEG_SM1_	0.65 ± 0.74	0.02–2.72	11
∼20 Hz MEG_SM1_ envelope	1.43 ± 1.56	0.02–4.84	12
By absolute change rate of the force			
MEG_SM1_	0.20 ± 0.30	0–1.20	6
∼20 Hz MEG_SM1_ envelope	0.34 ± 0.38	0–1.27	10
By heartbeats			
Force	4.1 ± 5.2	0.07–17	15
MEG_SM1_	1.2 ± 1.4	0–4.7	13
∼20 Hz MEG_SM1_ envelope	0.32 ± 0.37	0–1.4	8

### Impact of heartbeats on low-frequency coupling

**Figure 13.**
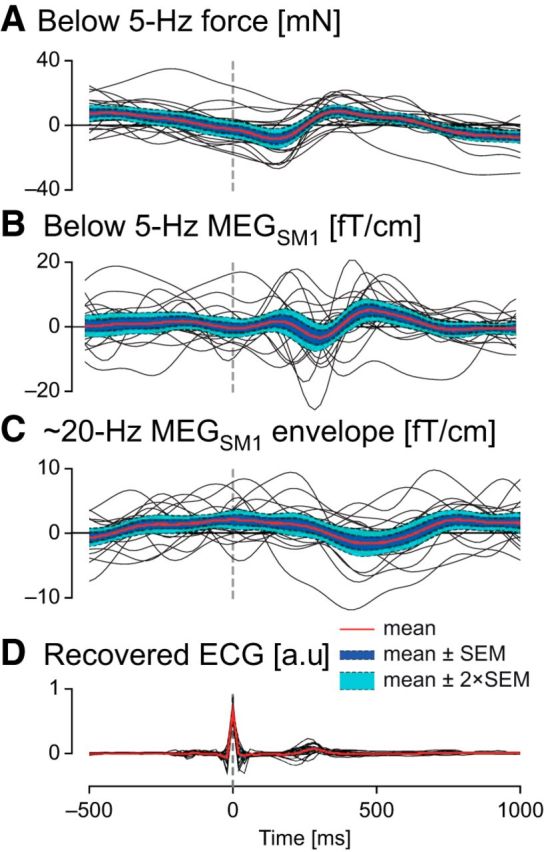
TRFs associated to heartbeat timing (***A***) of <5 Hz force, (***B***) of <5 Hz MEG_SM1_, (***C***) of ∼20 Hz MEG_SM1_ envelope, and (***D***) (for illustrative purpose) of ECG signals recovered from unprocessed MEG magnetometer data. In this latter case, the optimal ridge value was λ = 1. The plots display individual TRFs (black traces) as well as the mean TRF across subjects (red trace) ±1 SEM (blue area) and ±2 SEM (cyan area).

**Figure 14.**
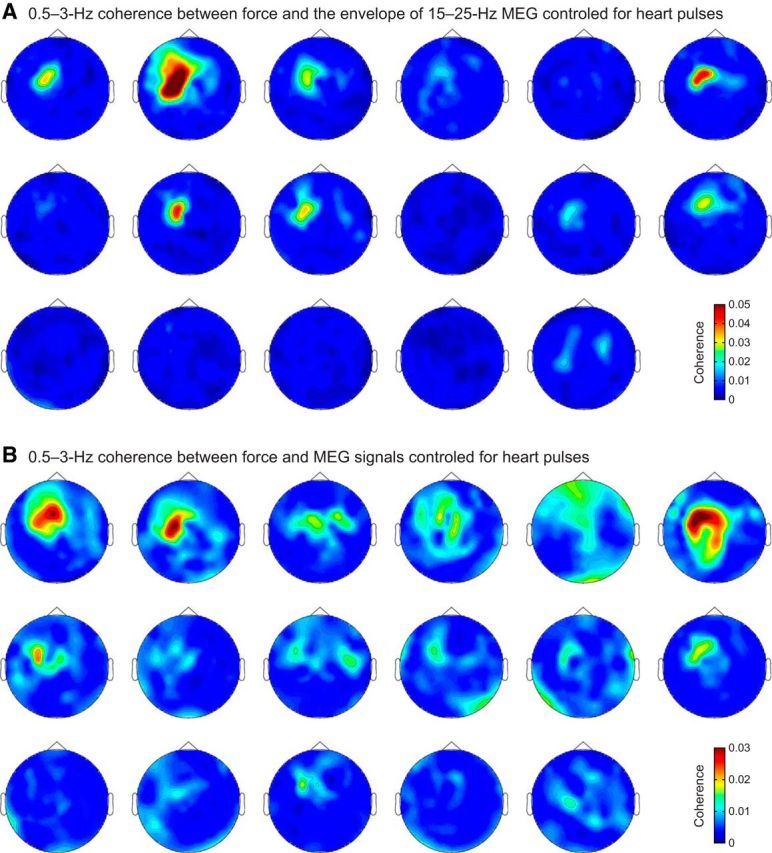
Sensor topography for the 0.5–3 Hz coherence of the force with (***A***) the envelope of 15–25 Hz MEG and (***B***) MEG signals controlled for heart pulses. Subjects are ordered as in Figure 7.

As the coupling we have just outlined occurs at frequencies overlapping with heart rate and its harmonics, we studied in detail the impact of heartbeats on force and brain signals. Figure 13 presents the TRFs of force, MEG_SM1_, and ∼20 Hz MEG_SM1_ envelope associated with heartbeats. Time-locked to magnetocardiographic R peak, subjects' contraction force consistently reached a minimum at ∼200 ms and was increased by, on average, 17 mN (which corresponds to a movement of ∼1 μm) at ∼400 ms. These timings are consistent with known delays between the electric and ballistocardiographic signals (Kim et al., 2016). No consistent trend was visible on subjects' MEG_SM1_ and ∼20 Hz MEG_SM1_ envelope responses to heartbeats. Overall, the TRFs explained only ∼4% of the variance of the 0.5–5 Hz content of force, and even less of that of MEG_SM1_ (∼1%) and ∼20 Hz MEG_SM1_ envelope (∼0.3%; Table 4). Importantly, the proportion of force variance explained by heart pulses was not predictive of the level of 0.5–3 Hz coherence between force and ∼20 Hz MEG_SM1_ envelope (*r* = 0.16, *p* = 0.53; Spearman correlation) or between force and MEG_SM1_ signals (*r* = 0.076, *p* = 0.77; Spearman correlation). Finally, the coherence in the sensorimotor sensors was mainly preserved when coherence between force and brain signals was controlled for heartbeats (Fig. 14, in comparison with Fig. 11). Indeed, at the MEG_SM1_ sensor, the ratio of partial coherence controlled for heartbeats to regular coherence was 0.97 ± 0.12 (mean ± SD; range 0.59–1.08) for coherence between force and ∼20 Hz MEG_SM1_ envelope, and 0.84 ± 0.20 (mean ± SD; range 0.21–1.02) for coherence between force and MEG_SM1_. Contrastingly, controlling for heartbeats led to strikingly damped coherence in the lowest planar sensors of the helmet (Fig. 14, in comparison with Fig. 11), which are known to be most affected by magnetocardiographic artifacts (Jousmäki and Hari, 1996). Indeed, at the sensor of maximum coherence with force among the 14 lowest-situated sensors of the helmet, the ratio of coherence controlled for heart pulses to regular coherence was 0.70 ± 0.35 (mean ± SD; range 0.10–1.02) for coherence between force and ∼20 Hz MEG envelope, and 0.61 ± 0.33 (mean ± SD; range 0.06–1.07) for coherence between force and MEG. Thus, the heartbeat artifacts contribute minimally to the 0.5–3 Hz coherence occurring between force and SM1 activity.

Because heartbeats consistently modulated force signals, we next tackled the question of whether these modulations would play a prominent physiological role in the 0.5–3 Hz coherence between force and SM1 activity. To that aim, we compared the different coherence estimates at f_heart-rate_ and f_off-heart-rate_. The mean coherence between force and MEG_SM1_ signals at f_heart-rate_ (0.021 ± 0.009) was ∼20% higher than that at f_off-heart-rate_ (0.018 ± 0.007), but this difference did not reach statistical significance (*p* = 0.08; Wilcoxon test). This result is in contrast with the finding that coherence between heartbeat and MEG_SM1_ signals was ∼3 times higher at f_heart-rate_ (0.025 ± 0.025) than at f_off-heart-rate_ (0.008 ± 0.010; *p* = 0.0006) and that the coherence between heartbeat and force signals was ∼4 times higher at f_heart-rate_ (0.049 ± 0.049) than at f_off-heart-rate_ (0.013 ± 0.015; *p* = 0.0003). Hence, we can conclude that, beyond heartbeats, there exists a prominent and independent coupling between force and SM1 activity at 0.5–3 Hz.

## Discussion

Our data favor the view that ∼20 Hz cortex–muscle coherence observed during isometric contraction builds on the presence of ∼20 Hz SM1 oscillations and needs not rely on feedback from the periphery. More importantly, our data suggest that effective cortical proprioceptive processing operates at lower, <3 Hz frequencies, even when the motor task does not involve voluntary movements. In other words, during steady isometric contraction, small fluctuations in muscle contraction are processed similarly as larger fluctuations occuring during continuous movements (Kelso et al., 1998; O'Suilleabhain et al., 1999; Jerbi et al., 2007; Bourguignon et al., 2011, 2012; Piitulainen et al., 2013a, b) and slow tracking movements (Dipietro et al., 2011; Hall et al., 2014).

### Pervasiveness of <3 Hz SM1 fluctuations

Based on our findings, we suggest the following mechanism for the maintenance of stable muscle contraction: The summed activity of proprioceptors sensitive to the force or its change rate (possibly in comparison with expected proprioceptive feedback) (Scott, 2012) modulates population-level SM1 excitability and the ∼20 Hz SM1 oscillations to compensate their cause. This homeostatic mechanism would directly account for how the brain gets informed about the low-frequency (<3 Hz) content of force fluctuations, which is the strongest and most relevant feedback signal, and needs to be continuously regulated to maintain a stable contraction. This interpretation emphasizes the importance of somatosensory feedback to control motor actions, in line with the influential view that the brain acts as a feedback controller for motor actions (Todorov and Jordan, 2002; Scott, 2004, 2012). Moreover, our finding sheds light on the type of signals the brain relies on to operate the way feedback controllers do.

Our MEG results do not tell whether increases and decreases of force were processed by identical, overlapping, or distinct neuronal populations. On the basis of animal neurophysiology, however, it is likely that the situation is mixed and complex because SM1 neurons can either increase or decrease their firing rate in response to force increase, or even display a mixed response pattern of, for example, phasic increase followed by tonic decrease (Wannier et al., 1991). Nevertheless, our finding that, during static contraction, SM1 signals were driven by the absolute change rate of the force rather than by the force itself is in line with and generalizes the previous observation that SM1 signals are also phase-locked to the absolute wrist velocity during slow tracking movements (O'Suilleabhain et al., 1999). Furthermore, SM1 signals are also triggered by the proprioceptive information during continuous movements (Kelso et al., 1998; O'Suilleabhain et al., 1999; Jerbi et al., 2007; Bourguignon et al., 2011, 2012; Piitulainen et al., 2013a, b) and slow tracking movements (Dipietro et al., 2011; Hall et al., 2014). This observation underlies the pervasiveness of <3 Hz SM1 fluctuations that appear to reflect the proprioceptive information not only during movements but also during steady contractions. Accordingly, these <3 Hz SM1 fluctuations may represent one of the necessary elements used by the brain to achieve accurate motor control. Direct cortical recordings (with, e.g., electrocorticography) are however needed in the future to clarify the respective contributions to the <3 Hz coupling within the different sensorimotor areas that we grouped here into an SM1 cortex.

Still, the slow fluctuations of the ∼20 Hz SM1 rhythm appeared to relate not only to somatosensory feedback, but also to activity that precedes changes in the force. Our results indeed revealed that the ∼20 Hz SM1 rhythm increased ∼200 ms before a change in the force, and that it was blocked ∼100 ms after the change. Enhancement of the ∼20 Hz SM1 rhythm has been suggested to indicate inhibition or deactivation of the motor cortex (Jasper and Penfield, 1949). The reason why the ∼20 Hz SM1 rhythm is modulated with such dynamics can only be speculated. The possible associated effects include transient loss of fine-motor control (e.g., due to attentional reallocation), which generates proprioceptive feedback and/or correction of the ongoing motor plan.

### Impact of heartbeats on force and brain signals

Heartbeats were associated with force changes of ∼17 mN, which explained ∼4% of the variance of slow force fluctuations (0.5–5 Hz). This finding is in line with a previous report that cardiovascular activity impacts contraction force during weak isometric finger contractions in the 0–6 Hz range among other frequencies (Sosnoff et al., 2011).

Still, heartbeats seemed to have only a limited impact on the coupling between <3 Hz force fluctuations and MEG signals. Indeed, using partial coherence to control for ECG signals attenuated the coupling with force at sensorimotor sensors by <20% while it effectively removed heartbeat-related artifacts in the lowest sensors of the helmet; these planar sensors are typically the most contaminated by the cardiac artifacts (Jousmäki and Hari, 1996). Moreover, coherence at f_off-heart-rate_ was <20% lower than coherence at f_heart-rate_. These converging values indicate that heartbeats contributed to <20% of the magnitude of the coupling between <3 Hz force fluctuations and MEG signals. This percentage of coherence relates to both heartbeat artifacts in MEG signals and genuine physiological modulation of the force by the heartbeats.

### Generation mechanism for the ∼20 Hz cortex–muscle coherence

Previous studies have suggested a central role for the ∼20 Hz SM1 oscillations in both encoding the motor command and processing the resulting proprioceptive feedback (Riddle and Baker, 2005; Baker, 2007; Witham et al., 2011; Aumann and Prut, 2015). Two action mechanisms have been hypothesized. First, the ∼20 Hz cortex–muscle coherence could arise because the SM1 cortex sends pulsed output and monitors the resulting afferent signals to probe the state of the periphery (Mackay, 1997; Baker, 2007; Witham et al., 2011). The second proposed mechanism posits that the ∼20 Hz cortex–muscle coherence reflects the integration of afferent information into motor commands to promote a stable motor state (Gilbertson et al., 2005; Androulidakis et al., 2006, 2007; Baker, 2007; Witham et al., 2011). However, our data challenge both these views and suggest that ∼20 Hz cortex–muscle coherence builds on the presence of ∼20 Hz SM1 oscillations and needs not rely on feedback from the periphery. We therefore propose below a hypothetical generation mechanism of ∼20 Hz cortex–muscle coherence.

In monkeys maintaining isometric contraction, the spiking activity of pyramidal-tract neurons is coupled with local field potentials in the SM1 cortex at both ∼10 and ∼20 Hz (Baker et al., 2003). In other words, the excitability of the pyramidal neurons relates to the phase of the ∼10 and ∼20 Hz SM1 oscillations; and consequently, the common pyramidal output tends to structure according to these oscillations. However, cortex–muscle coherence seldom peaks at ∼10 Hz, which speaks for the existence of a blocking mechanism that prevents the excitability of motoneuron pools from oscillating at ∼10 Hz despite the presence of these frequencies in the corticospinal drive (Baker et al., 2003). Such a blocking was argued to be important to prevent excess ∼10 Hz physiological tremor (Baker et al., 2003). Given the close-to-harmonic relationship between ∼10 and ∼20 Hz SM1 oscillations, we simply suggest that the same mechanism tends to block ∼20 Hz oscillatory motor output as well. The ∼20 Hz cortex–muscle coherence would then emerge due to nonperfect blocking of the ∼20 Hz oscillatory input to the spinal α motoneurons.

### Perspectives for the ∼20 Hz cortex–muscle coherence

Abnormally high ∼20 Hz cortex–muscle coherence has been observed in patients suffering from dysfunction of the sensorimotor system, especially of myoclonic disorders (Timmermann et al., 2001; Raethjen et al., 2002; Silén et al., 2002; Caviness et al., 2003; Grosse et al., 2003; Kristeva et al., 2004). Given the very strong association between the magnitude of cortex–muscle coherence and the burstiness of ∼20 Hz force or EMG signals, we suggest that the observed abnormally high cortex–muscle coherence primarily reflects altered functioning at the level of the periphery, which would be seen (but has not yet been addressed) as high burstiness of both force and EMG due to the myoclonic disorder. Thus, a future alternative means to estimate the level of cortex-muscle coupling would be to quantify the burstiness (with the CoV-E) of both force and EMG; these signals are indeed easily accessible, as they only require measurement of contraction force or muscle activity, with no need to record brain activity.

Finally, our findings provide a novel insight into a so far unresolved puzzle about the cortex–muscle coherence, that is, its great interindividual variability, even in healthy subjects. It was reported that the maximum level of cortex–muscle coherence correlates positively with force CoV and force power in the α and β bands, but the Pearson correlation coefficients were <0.65 (Ushiyama et al., 2017). Here, we demonstrate a positive correlation between maximal cortex–muscle coherence and CoV-E of ∼20 Hz force and EMG fluctuations characterized by Pearson correlation coefficients of ∼0.9. Accordingly, the present study considerably clarifies the situation by revealing that individual levels of ∼20 Hz cortex–muscle coherence are strongly related to the “burstiness” of EMG and force signals at ∼20 Hz. In other words, the questions “why is the ∼20 Hz cortex-muscle coherence fraught with so high interindividual variability” and “why does the sensorimotor system of different individuals suppress differently the ∼20 Hz content of the motor command” are equivalent.
